# Triggering Receptors Expressed on Myeloid Cells 2 Promotes Corneal Resistance Against *Pseudomonas aeruginosa* by Inhibiting Caspase-1-Dependent Pyroptosis

**DOI:** 10.3389/fimmu.2018.01121

**Published:** 2018-05-25

**Authors:** Wenting Qu, Yi Wang, Yongjian Wu, Yiting Liu, Kang Chen, Xi Liu, Zhengyu Zou, Xi Huang, Minhao Wu

**Affiliations:** ^1^Program of Pathobiology and Immunology, Fifth Affiliated Hospital, Zhongshan School of Medicine, Sun Yat-sen University, Guangdong, China; ^2^Key Laboratory of Tropical Diseases Control, Ministry of Education, Sun Yat-sen University, Guangzhou, China; ^3^Department of Laboratory Medicine, Zhongshan Hospital of Sun Yat-sen University, Zhongshan, China; ^4^Guangdong Engineering & Technology Research Center for Disease-Model Animals, Sun Yat-sen University, Guangzhou, China

**Keywords:** *Pseudomonas aeruginosa*, triggering receptors expressed on myeloid cells 2, corneal infection, pyroptosis, inflammation, bacterial killing

## Abstract

Triggering receptors expressed on myeloid cells 2 (TREM2) is a novel cell surface receptor and functions as an immunomodulatory receptor in infectious diseases. In this study, we investigated the function and regulatory mechanism of TREM2 in *Pseudomonas aeruginosa* (*P. aeruginosa*) keratitis. We found that *P. aeruginosa* keratitis was more severe in *Trem2*^−/−^ versus wild type C57BL/6 mice as indicated by the increased clinical scores, bacterial load, and cornea pathology. The exacerbated disease progression caused by TREM2 deficiency was associated with boosted activation of caspase-1 and subsequent pyroptosis as well as increased expression of IL-1β. In addition, blockage of pyroptosis by caspase-1 inhibitor not only recovered the severe cornea pathology developed in *Trem2*^−/−^ mice but also restored the *P. aeruginosa* clearance suppressed by TREM2 deficiency. Our study demonstrated that TREM2 promotes host resistance against *P. aeruginosa* keratitis by inhibiting caspase-1-dependent pyroptosis, which provides new insights of TREM2-mediated anti-bacterial immunity.

## Introduction

*Pseudomonas aeruginosa* (*P. aeruginosa*) is one of the most common Gram-negative bacteria that cause diverse opportunistic infectious diseases, including keratitis in extended wear contact lens users (1). Clinically, *P. aeruginosa* keratitis progresses rapidly and results in severe corneal damage, including perforation and vision loss (1, 2). Both bacteria virulence factors and host inflammatory mediators contribute to the destruction of the cornea after infection (3).

Upon *P. aeruginosa* infection, diverse pathogen-associated molecular patterns were specifically recognized by corresponding pattern recognition receptors (PRRs), such as toll-like receptors (TLRs) (4, 5) and Nod-like receptors (NLRs) (6). In *P. aeruginosa*-induced keratitis (corneal infection), the TLR-induced inflammatory response promotes bacteria clearance and repairs injured tissues. However, if out of control, the excessive inflammation also leads to tissue damage and corneal ulceration. Except for TLRs, *P. aeruginosa* infection was able to activate certain numbers of the NLR family, such as the NLRC4 (7) and NLRP3 (8), which function as core receptor molecules to initiate the assembly of multi-protein complexes named inflammasomes. Upon stimulation, the NLRs recruit the adaptor protein ASC *via* PYD–PYD domain association, and ASC further recruits precursors of caspase-1 through CARD–CARD domain interaction. After clustering and autocleavage, the activated caspase-1 is able to cleave the pro-IL-1β and pro-IL-18 into their mature forms, as well as initiate a type of rapid inflammatory cell death termed pyroptosis (9–11). Pyroptosis has been reported to play an important role in promoting the elimination of several intracellular bacteria, including *Legionella pneumophila* (12) and *Francisella tularensis* (13). Whereas in the setting of acute *P. aeruginosa* pneumonia, inhibition of inflammasome signaling significantly enhanced bacterial clearance *via* reducing cell death (14). However, whether pyroptosis is involved in the pathogenesis of *P. aeruginosa* keratitis is still uncertain.

Triggering receptors expressed on myeloid cells 2 (TREM2) is a novel member of PRRs and broadly existed on the surface of mononuclear phagocytes, including macrophages, microglia, and osteoclast precursors (15). TREM2 has been reported as an immune regulator and exerts important functions in bacteria clearance (16, 17). Recently, Holtzman’s group reported that TREM2 suppressed the apoptosis of lung macrophages during Sendai virus infection, then led to chronic inflammatory diseases (18). Colonna’s lab found that in Alzheimer’s disease (AD) patients and the 5xFAD transgenic murine model, deficiency in TREM2 caused autophagy of microglia *in vivo* (19). These results indicated the correlation between TREM2 and programmed cell death. Our previous studies demonstrated that TREM2 protected *P. aeruginosa*-infected corneas of BALB/c mice (which are Th2 responders and *P. aeruginosa*-resistant strain) by suppressing corneal inflammation and bacterial load (20). However, whether inflammasomes and pyroptosis are involved TREM2-mediated host defense against *P. aeruginosa* is still unclear.

In this study, we explored the relationship between TREM2 and caspase-1-dependent pyroptosis, and investigated the role of pyroptosis in *P. aeruginosa* keratitis using TREM2-deficient versus wild type C57BL/6 mice (which is a *P. aeruginosa-*susceptible strain). Our *in vivo* results indicated that TREM2 suppressed activation of caspase-1 and subsequent pyroptosis, thereby inhibiting cornea pathology as well as promoting the clearance of *P. aeruginosa*. These findings provide a better understanding of TREM2-mediated pyroptosis and bacterial eradication.

## Materials and Methods

### Mice and Reagents

Wild type (WT) C57BL/6 mice were purchased from the experimental animal center of Sun Yat-sen University. *Trem2*^−/−^ C57BL/6 mice were generously provided by Marco Colonna (Washington University School of Medicine). *P. aeruginosa* (*P. aeruginosa*; strain 19660) was purchased from the American Type Culture Collection (ATCC; Manassas, VA, USA). Heat-killed *P. aeruginosa* (HK-PA) was prepared according to previous study (21). *P. aeruginosa* (ATCC 19660) was killed by heating 100 μl aliquots of bacteria solution [10^8^ colony forming units (CFU)/ml] at 65°C for an hour. A sample of heat-killed PA was grown on PIA (BD Difco Laboratories) plates, and no live PA was observed after incubation at 37°C for 24 h. Ac-YVAD-CMK (caspase-1 inhibitor, Cat: SML0429), DMSO (Cat: D2650), and anti-β-actin (clone: AC-15; Cat: A1978) were purchased from Sigma-Aldrich (St. Louis, MO, USA). Anti-mouse caspase-1 (p20) (clone: Casper-1; Cat: AG-20B-0042) and anti-mouse NLRP3 (clone: Cryo-2; Cat: AG-20B-0014) were ordered from Adipogen (San Diego, CA, USA). Anti-ASC (clone: N-15; Cat: sc-22514-R) was ordered from Santa Cruz biotechnology (San Diego, CA, USA). Anti-GSDMD (clone: EPR19828; Cat: ab209845) and Anti-TREM2 (clone: 6EP9; Cat: ab125117) were purchased from Abcam (Cambridge, MA, USA). Anti-caspase-11 (clone: 17D9; Cat: #14340) was purchased from Cell Signaling Technology (Danvers, MA, USA). Secondary antibodies against mouse and rabbit were obtained from Bio-Rad (Hercules, CA, USA).

### Ocular Infection and Clinical Examination

The left cornea of the 6-week-old female WT or *Trem2*^−/−^ B6 mice was infected by *P. aeruginosa* (ATCC 19660) as described before (22). Eyes were examined at 1, 3, and 5 days postinfection (p.i.) or at times described below. Corneal disease was graded using an established scale (23): 0, clear or slight opacity partially or fully covering the pupil; +1, slight opacity partially or fully covering the anterior segment; +2, dense opacity partially or fully covering the pupil; +3, dense opacity covering the entire anterior segment; and +4, corneal perforation or phthisis. A clinical score was recorded for each mouse after infection for statistical comparison of disease severity. Photography with a slit lamp was used to confirm and document the disease response. Animals were treated humanely and in compliance with the ARVO Statement for the Use of Animals in Ophthalmic and Vision Research.

### Isolation of Bone Marrow-Derived Macrophages (BMDMs) and Cell Culture

Mouse BMDMs were isolated and differentiated as reported previously (20). In brief, WT or *Trem2*^−/−^ mice were sacrificed and the bone marrow in femurs was flushed out and cultured in DMEM containing 10% FBS and 30% (vol/vol) L-929 fibroblast-conditioned medium as a source of macrophage colony-stimulated factor. BMDMs were obtained as a homogeneous population of adherent cells after 7 days culture. The BMDM purity was routinely >95% as assessed by flow cytometry. A6(1) corneal epithelial cell line was a gift from Dr. Peggy Zelenka (National Eye Institute/NIH) and cultured following their protocol (24). The A6(1) cells were derived from corneal epithelia of the 14-day-old immortomouse (Charles River Laboratories, Wilmington, MA, USA), then conditionally immortalized by a temperature sensitive SV40 T-antigen, under the control of an IFN-γ inducible promoter. Cells were cultured in Epilife™ media, supplemented with corneal epithelial growth supplement, interferon IFN-γ (5 units/ml), 1% penicillin–streptomycin, 1% l-glutamine, and 20% (v/v) FBS (all from Invitrogen) at the permissive temperature of 33°C, in a humidified atmosphere of 95% air and 5% CO_2_. For *in vitro* assays, subconfluent cultures were transferred to the nonpermissive temperature of 37°C and cultured in the same media, but without IFN-γ.

### Real-Time PCR

Mice were sacrificed on 1 and 5 days p.i. and infected corneas of WT and *Trem2*^−/−^ B6 mice (*n* = 5/group/time) were harvested. Total RNA was isolated from individual corneas using TRIzol (Invitrogen, Carlsbad, CA, USA) as described previously (23). Total RNA was quantitated using a NanoDrop 2000C Spectrophotometers (Thermo Scientific, West Palm Beach, FL, USA). 1 µg of total RNA was reversely transcribed to produce cDNA by using RevertAid First Strand cDNA synthesis kit (Thermo Fisher Scientific, Waltham, MA, USA), and then amplified using SYBR Green Master Mix (Thermo Fisher Scientific, Waltham, MA, USA) following the manufacturer’s protocol. Quantitative real-time PCR reactions were performed using the CFX96 Real-Time PCR System (Bio-Rad, Hercules, CA, USA). The average threshold cycle (CT) values of samples were normalized to CT of β-actin gene. The relative expression was determined by the 2^−ΔΔCT^ method. Primer sequences are listed in Table S1 in Supplementary Material.

### Western Blot

To detect the corneal expression of NLRP3, ASC, caspase-11, caspase-1, and GSDMD, whole corneas (*n* = 5/group/time) were collected and pooled from normal uninfected and infected B6 mouse eyes at 1 and 5 days p.i. Pooled corneas were lysed and homogenized using a 1-ml glass tissue homogenizer in lysis buffer containing 1 mM phenylmethylsulfonyl fluoride, 1% (vol/vol) protease inhibitor cocktail, and 1 mM DTT (all from Sigma, St. Louis, MO, USA). Then, protein concentration of the supernatant was determined by Quick Start Bradford protein assay (Bio-Rad). 20 µg of each sample was loaded, separated on 12% SDS-PAGE, and then transferred to a supported nitrocellulose membrane (Pall Life Sciences, Ann Arbor, MI, USA). After blockage, blots were incubated overnight with the respective primary antibodies at 4°C, followed by incubation with appropriate HRP-conjugated secondary antibodies at room temperature for 1 h. Finally, blots were visualized with New-SUPER ECL (KeyGEN, Nanjing, China) according to the manufacturer’s protocol.

### Hematoxylin-Eosin (HE) Staining and Immunohistochemistry

Infected eyes were enucleated (*n* = 3/group/time) at 1 and 5 days p.i. from WT and *Trem2*^−/−^ B6 mice, embedded in Tissue-Tek OCT compound (Miles, Elkhart, IN, USA) and frozen in liquid nitrogen. 8 µm thick sections were cut and mounted to glass slides. For histopathology, sections were hematoxylin-eosin (HE) stained as described by others (25). Immunohistochemical staining was performed with the UltraSensitive SP Immunodetection Kit (Maixin, Inc., Fuzhou, China) according to the manufacturer’s instructions. Primary antibodies rabbit anti-mouse capase-1 (p10) (clone: M-20; Cat: sc-514) were purchased from Santa Cruz. All sections were visualized with a Carl Zeiss microscope (Carl Zeiss Inc.).

### Terminal Deoxynucleotidyl Transferase-Mediated Uridine 5′-Triphosphate-Biotin Nick End Labeling (TUNEL) Staining

Infected eyes from the WT versus *Trem2*^−/−^ B6 mice (*n* = 3/group/time) were enucleated at 5 days p.i. for TUNEL staining with a terminal deoxynucleotidyl transferase (TdT) kit (Promega, Madison, WI, USA) following the manufacturer’s instruction. Eyes were fixed in a 3.7% formaldehyde solution (Sigma) and embedded in paraffin. 8 µm thick sections were cut, deparaffinized, rehydrated, and rinsed with DNase-free water (Invitrogen). Sections were permeabilized using proteinase K solution (20 µg/ml, Sigma) for 15 min and then fixed again using 3.7% formaldehyde solution (Sigma). Each section was incubated with TdT incubation buffer, which contains 45 µl equilibration buffer, 5 µl nucleotide mix, and 1 µl TdT enzyme at 37°C for 1 h to label the DNA nick ends, and then incubated with 4,6-diamino-2-phenyl indole (DAPI, 1:10,000, Sigma) for nuclear staining. All sections were visualized with a Carl Zeiss microscope (Jena, Germany).

### Bacterial Plate Counts

Corneas from WT versus *Trem2*^−/−^ B6 mice (at 1 and 5 days p.i.) were harvested (*n* = 5/group/time) and the number of viable bacteria was quantitated as described before (26). Individual corneas were homogenized in normal saline solution containing 0.25% BSA. Serial 10-fold dilutions of the samples were plated on PIA (BD Difco Laboratories) in triplicate and plates were incubated overnight at 37°C. Results are reported as 10^5^ CFU per cornea ± SEM.

### Enzyme-Linked Immunosorbent Assay (ELISA)

Corneas from WT versus *Trem2*^−/−^ B6 mice were individually collected (*n* = 5/group/time) at 1 and 5 days p.i. Corneas were homogenized in 0.5 ml of PBS with 0.1% Tween 20. All samples were centrifuged at 13,000 rpm for 5 min and an aliquot of each supernatant was assayed in duplicate for IL-1β protein by using ELISA kits from BD Biosciences (San Jose, CA, USA) following the manufacturer’s instructions. The reported sensitivity of these assays is <3.0 pg/ml for IL-1β.

### Flow Cytometric Detection of Activated Caspase-1

Individual corneas from WT and *Trem2*^−/−^ B6 mice (*n* = 5/group) were harvested at 5 days postinfection. Individual corneas were kept in sterile tubes containing 1 ml Liberase TL (Roche, Indianapolis, IN, USA, 2.5 mg/ml), followed by incubation at 37°C for 45 min. At the end of the incubation period, samples were triturated using a 2 ml syringe plunger, and passed through a 70 µm cell strainer. Finally, the single-cell suspension was washed with 1 ml RPMI 1640 containing 10% FBS and was pelleted at 500 × *g* for 8 min in a refrigerated centrifuge. Caspase-1 activation was detected in the corneas using a FAM-FLICA™ caspase-1 assay kit following the manufacturer’s instructions (Immunochemistry Technologies, Bloomington, MN, USA). Briefly, single-cell suspensions of infected corneas from WT and *Trem2*^−/−^ B6 mice were incubated with FAM–FLICA caspase-1 at 37°C, 5% CO_2_ for 45 min. At the end of the incubation period, cells were washed twice with 1× wash buffer provided in the kit. Cell surface staining for CD11b, Gr1, F4/80, and CD11c molecules was carried out and after samples were immediately acquired using a LSRFortessa flow cytometer (Beckton Dickinson, San Jose, CA, USA). Data were analyzed using FlowJo software and presented as mean fluorescence intensity ± SEM. The following antibodies were used for cell surface staining: FITC-conjugated rat anti-mouse CD11b (M1/70, 1:200), APC-conjugated rat anti-mouse F4/80 (BM8, 1:200), PerCP-Cy5.5-conjugated rat anti-mouse Gr1 (RB6-8C5, 1:200), and PE-Cy7-conjugated rat anti-mouse CD11c (N418, 1:200). All antibodies were purchased from BD Biosciences (San Jose, CA, USA), eBiosciences (Waltham, MA, USA), or BioLegend (San Diego, CA, USA).

### Immunoprecipitation

Individual uninfected and infected corneas from WT B6 mice were harvested at 5-day postinfection. Pooled corneas were lysed and homogenized using a 1-ml glass tissue homogenizer in Pierce IP lysis buffer. BMDMs from WT B6 mice were treated with HK-PA or stimulated with nigericin. After treatment or stimulation, cells were lysed with Pierce IP lysis buffer. The supernatant of cell lysates was incubated with protein A and anti-mouse TREM2 antibody overnight at 4°C to pull down protein complexes. The protein complexes were pelleted and washed three times with IP washing buffer. The immunoprecipitated protein complex was analyzed by immunoblot with anti-TREM2, anti-NLRP3, and anti-caspase-1. And β-actin, TREM2, NLRP3, and caspase-1 were also detected in input cell lysates by western blot analysis.

### Statistical Analysis

The differences between two groups were analyzed by using an unpaired two-tailed Student’s *t*-test. Mann–Whitney *U* test was used to determine the difference in clinical score between two groups. Differences were considered statistically significant when the *P* value was <0.05.

## Results

### TREM-2 Promoted Host Resistance Against *P. aeruginosa* Keratitis

**Figure 1:**
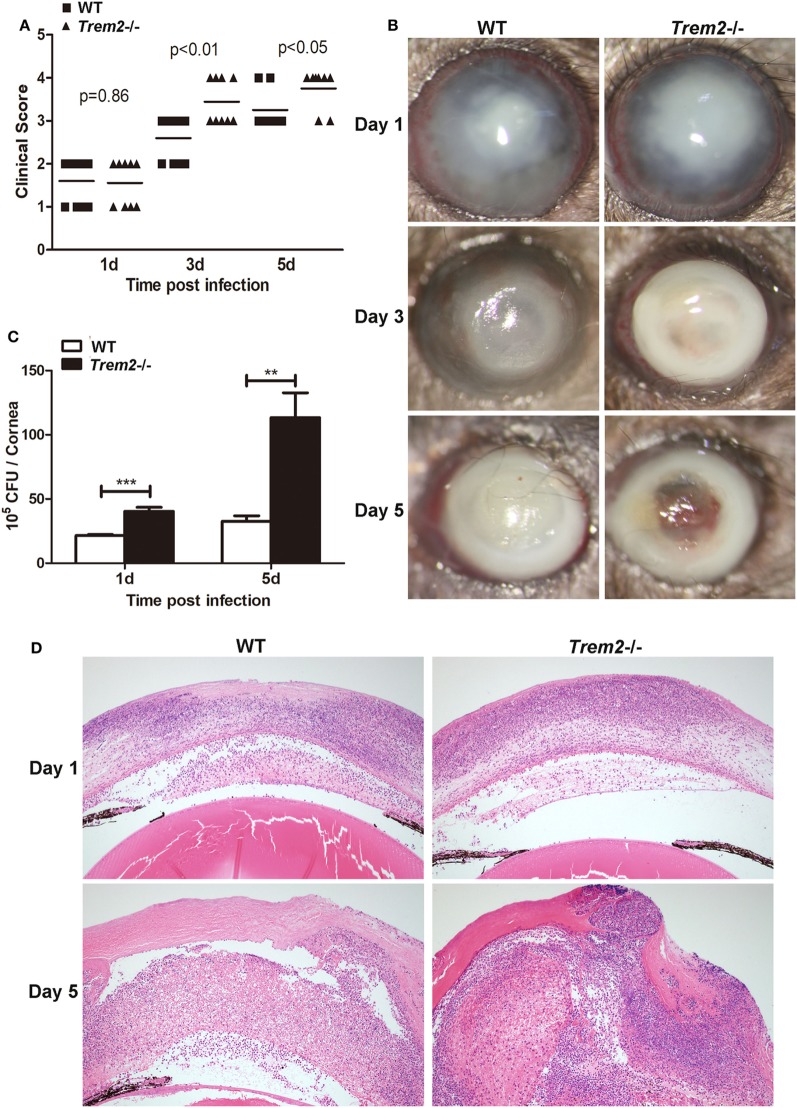
Triggering receptors expressed on myeloid cells 2 (TREM-2) promoted host resistance against *Pseudomonas aeruginosa* keratitis. *Trem2*^−/−^ and wild type (WT) C57BL/6 mice were infected with *P. aeruginosa*. **(A)** Clinical score was recorded for each cornea at 1, 3, and 5 days after infection. **(B)** Representative slit-lamp photographs of infected eyes in WT (left) and *Trem2*^−/−^ (right) mice were taken 1, 3, and 5 days after infection. **(C)** Bacterial load in the infected corneas was examined by plate count assay in WT versus *Trem2*^−/−^ mice at 1 and 5 days after infection. **(D)** Hematoxylin and eosin staining was used to examine the histopathology of infected eyes in WT (left) and *Trem2*^−/−^ (right) mice at 1 and 5 days after infection. Magnification = 100×. Data are the mean ± SEM and represent three individual experiments each with 10 mice per group **(A,B)** or five mice per group **(C,D)**. *P < 0.05; **P < 0.01; ***P < 0.001.

To confirm the role of TREM-2 in the pathogenesis of *P. aeruginosa* keratitis, wild type (WT) and *Trem2*^−/−^ C57BL/6 mice were infected with *P. aeruginosa* routinely. Clinical scores were significantly higher in the corneas of *Trem2*^-/^mice compared with WT group at 3 and 5 days p.i. (Figure 1A). Representative photographs taken with a slit lamp of the infected corneas in WT (Figure 1B left) and *Trem2*^−/−^ (Figure 1B right) mice were taken to illustrate disease severity. TREM2 deficiency resulted in corneal perforation (grade = + 4, Figure 1B right on the bottom), while the pupil of infected WT mice was partially or fully covered with dense opacity (grade = + 3, Figure 1B left on the bottom) at 5 days p.i. Since the disease pathogenesis of *P. aeruginosa* keratitis depends on both bacterial virulence and host inflammation, we first assessed the effects of TREM2 on bacterial load. As shown in Figure 1C, TREM2 deficiency elevated the number of viable bacteria in the infected corneas at 1 and 5 days p.i. We further compared the corneal inflammation in WT and *Trem2*^−/−^ mice by enucleating the infected eyes for histopathology. Hematoxylin and eosin (H&E) staining results indicated that *Trem2*^−/−^ corneas (Figure 1D right) were more swollen, with more infiltrated inflammatory cells and exhibited more severe tissue destruction, in contrast to the WT corneas (Figure 1D left). Together, these results suggested that TREM2 promoted host resistance to *P. aeruginosa* corneal infection in C57BL/6 mice.

### TREM-2 Inhibited Pro-Inflammatory Cytokine Expression in *P. aeruginosa* Keratitis

**Figure 2:**
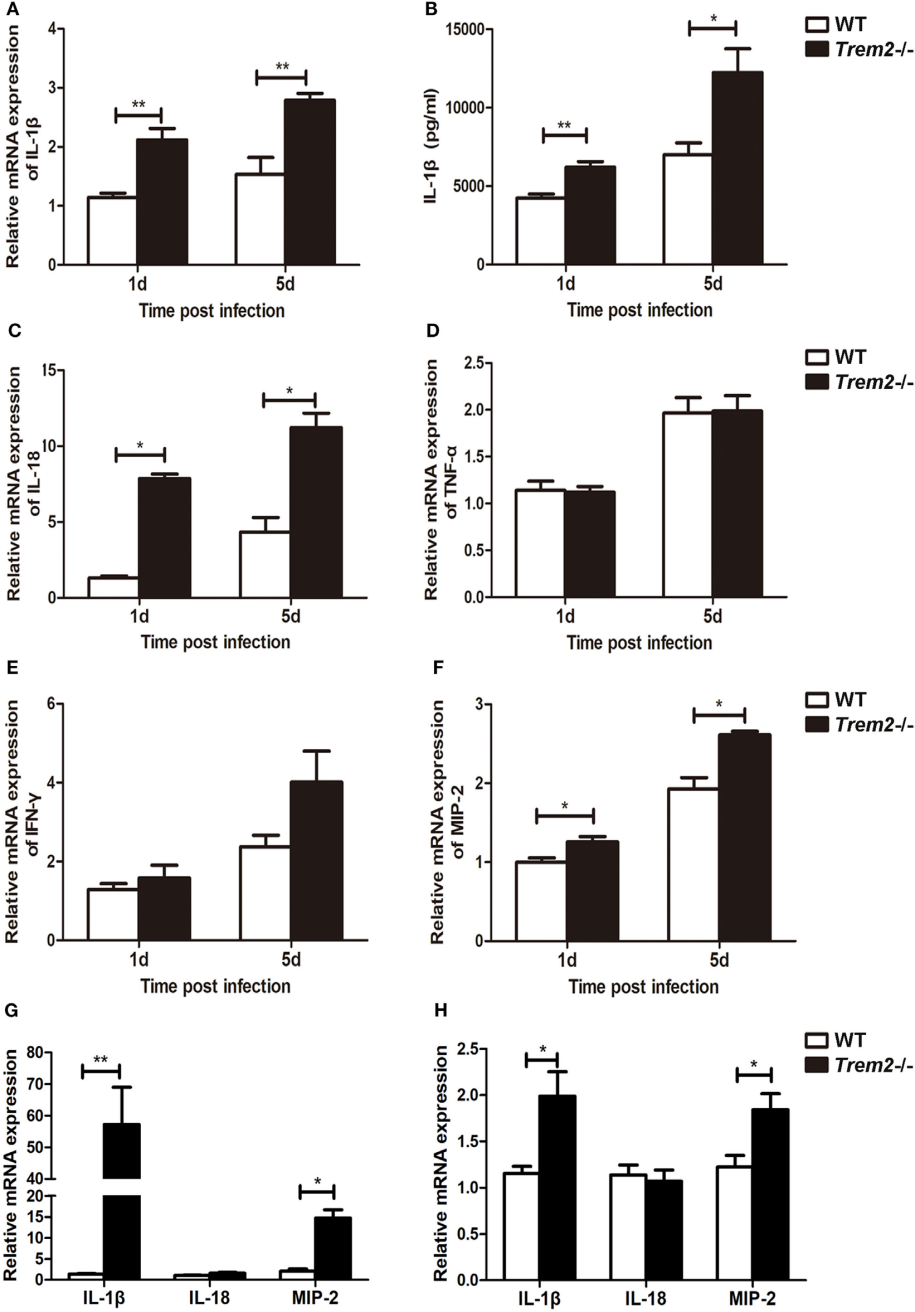
Triggering receptors expressed on myeloid cells 2 (TREM-2) inhibited pro-inflammatory cytokine expression after *Pseudomonas aeruginosa* infection. mRNA expression levels of pro-inflammatory cytokines, including IL-1β **(A)**, IL-18 **(C)**, tumor necrosis factor-alpha **(D)**, IFN-γ **(E)**, and MIP-2 **(F)** was examined by real-time PCR in infected wild type (WT) and *Trem2*^−/−^ B6 corneas at 1 and 5 days after infection. **(B)** Protein level of IL-1β in WT and *Trem2*^−/−^-infected B6 corneas was tested by enzyme-linked immunosorbent assay at 1 and 5 days after infection. mRNA expression levels of IL-1β, IL-18, and MIP-2 was examined by real-time PCR in heat-killed *P. aeruginos*a (HK-PA)-treated WT and *Trem2*^−/−^ B6 corneas at 5 days after treatment **(G)** and WT and *Trem2*^−/−^ BMDM **(H)** which were treatment with HK-PA at an MOI of 5 for 6 h. Data are the mean ± SEM and represent three individual experiments each with five mice per group. *P < 0.05; **P < 0.01.

As the inflammation was more severe in TREM2-deficient corneas (Figure 1D), we further determined the mechanism by which TREM-2 mediated the inflammatory response. We first checked the expression of pro-inflammatory cytokines and found that TREM2 deficiency markedly upregulated the mRNA levels of IL-1β (Figure 2A) and IL-18 (Figure 2C) as well as the protein levels of IL-1β (Figure 2B) at 1 and 5 days p.i. Expression and secretion of IL-1β can be promoted through either inflammasome-dependent or NF-κB pathway. However, the expression levels of tumor necrosis factor-alpha (TNF-α), a proinflammatory cytokine associated with NF-κB activation, were similar in these two groups (Figure 2D). Suggesting the increased IL-1β in *Trem2*^−/−^ corneas was largely inflammasome-dependent. Furthermore, corneas of *Trem2*^−/−^ mice appeared in higher expression of inflammasome-independent cytokines IFN-γ (Figure 2E) and MIP-2 (Figure 2F). To investigate whether the overall increase in these proinflammatory cytokines was due to the higher bacterial load in *Trem2*^−/−^ corneas, we inoculated WT and *Trem2*^−/−^ mice with HK-PA routinely. We found that expression of IL-1β and MIP-2 was also upregulated in *Trem2*^−/−^ corneas while expression of IL-18 was comparable between the two groups (Figure 2G). Moreover, we observed similar results when WT and *Trem2*^−/−^ BMDMs were treated with HK-PA (Figure 2H). These data indicated that TREM-2 downregulated the corneal inflammation *via* inhibiting expression of proinflammatory cytokine IL-1β and IL-18 in response to *P. aeruginosa* ocular infection.

### TREM-2 Inhibited the Activation of Caspase-1 and Subsequent Pyroptosis After *P. aeruginosa* Infection

**Figure 3:**
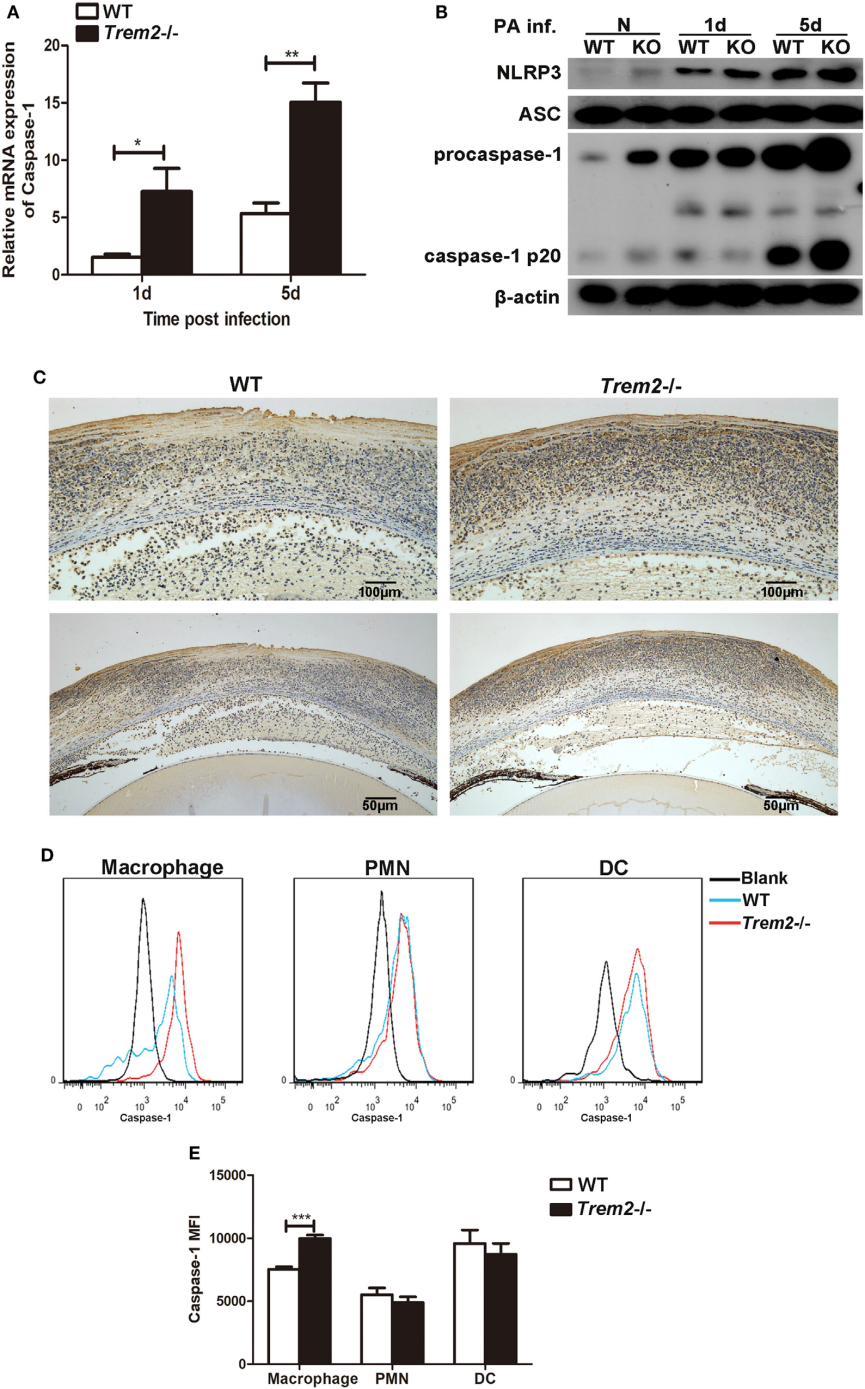
Triggering receptors expressed on myeloid cells 2 (TREM-2) inhibited the expression and activation of caspase-1 after *Pseudomonas aeruginosa* infection. mRNA expression level of caspase-1 **(A)** was examined by real-time PCR in infected wild type (WT) and triggering receptors expressed on myeloid cells 2 (*Trem2*)^−/−^ B6 corneas at 1 and 5 days after infection. **(B)** The protein levels of total and cleaved caspase-1, NLRP3, and ASC in infected WT and *Trem2*^−/−^ B6 corneas were detected with western blot at 1 and 5 days after infection. **(C)** Caspase-1 protein expression was also determined by using immune-histochemistry in WT (left) and *Trem2*^−/−^ (right) B6 corneas after *P. aeruginosa* infection. Magnification was 100× at top panel and 200× at bottom panel, respectively. **(D)** FAM-FLICA staining was used to detect activated caspase-1 in macrophages (left), polymorphonuclear neutrophils (middle), and dendritic cells (right) from the infected WT and *Trem2*^−/−^ corneas at 5 days postinfection. **(E)** The mean fluorescence intensity for activated caspase-1 was analyzed using FlowJo software. Data were the mean ± SEM and represent three individual experiments each with five mice per group. *P < 0.05; **P < 0.01.

As the expression and secretion of IL-1β were significantly increased in TREM2-deficient corneas after *P. aeruginosa* infection (Figures 2A–C), we further detected the expression and activation of caspase-1 in the corneas of WT and *Trem2*^−/−^ mice. Indeed, *Trem2*^−/−^ corneas appeared in higher mRNA expression of caspase-1 (Figure 3A). The expression level of caspase-1 was also higher in *Trem2*^−/−^ corneas and BMDMs after HK-PA treatment (Figure S1A in Supplementary Material). As full catalytic activity of caspase-1 requires autoproteolytic processing of procaspase-1, we also detected caspase-1 cleavage by Western blot. Results showed that *P. aeruginosa*-induced caspase-1 cleavage (caspase-1 p20) as well as procaspase-1 expression was significantly increased in *Trem2*^−/−^ corneas (Figure 3B). At the same time, the expression of NLRP3 was also higher in infected *Trem2*^−/−^ corneas, while ASC levels were comparable between the two groups before and after *P. aeruginosa* infection (Figure 3B). Caspase-1 expression and activation after *P. aeruginosa* infection were further confirmed by immune-histochemistry. Higher level of positive staining for caspase-1 (depicted as brown dots) was detected in the infected *Trem2*^−/−^ B6 corneas, mainly localizing in the corneal epithelium and stroma (Figure 3C). Since the noncanonical inflammasome activation mediated by caspase-11 can trigger both caspase-1-dependent and -independent production of IL-1β and IL-18, we also tested caspase-11 expression in the infected WT and *Trem2*^−/−^ corneas and found its expression was comparable between the two groups (Figure S1B in Supplementary Material). To further determine what cell type plays important role in the severe pathology mediated by TREM2 deficiency, we investigated the caspase-1 activation on the major cell subsets in the infected WT and *Trem2*^−/−^ corneas by flow cytometry. Our results showed that caspase-1 activation was enhanced in macrophages from *Trem2*^−/−^ when compared to those from WT corneas, while its activation in polymorphonuclear neutrophils (PMN) and dendritic cells (DC) was comparable (Figures 3D,E). In addition, we also tested the role of TREM2 in A6(1), which is a mouse corneal epithelial cell line. Our data indicated that knockdown TREM2 in A6(1) did not affect the expression of caspase-1 as well as inflammatory cytokine, such as IL- β and MIP-2 after HK-PA treatment (Figure S1C in Supplementary Material). These findings support the hypothesis that the regulatory function of TREM2 in *P. aeruginosa* keratitis is exerted in macrophages, not other cell types, including PMNs, DCs, or corneal epithelial cells.

**Figure 4:**
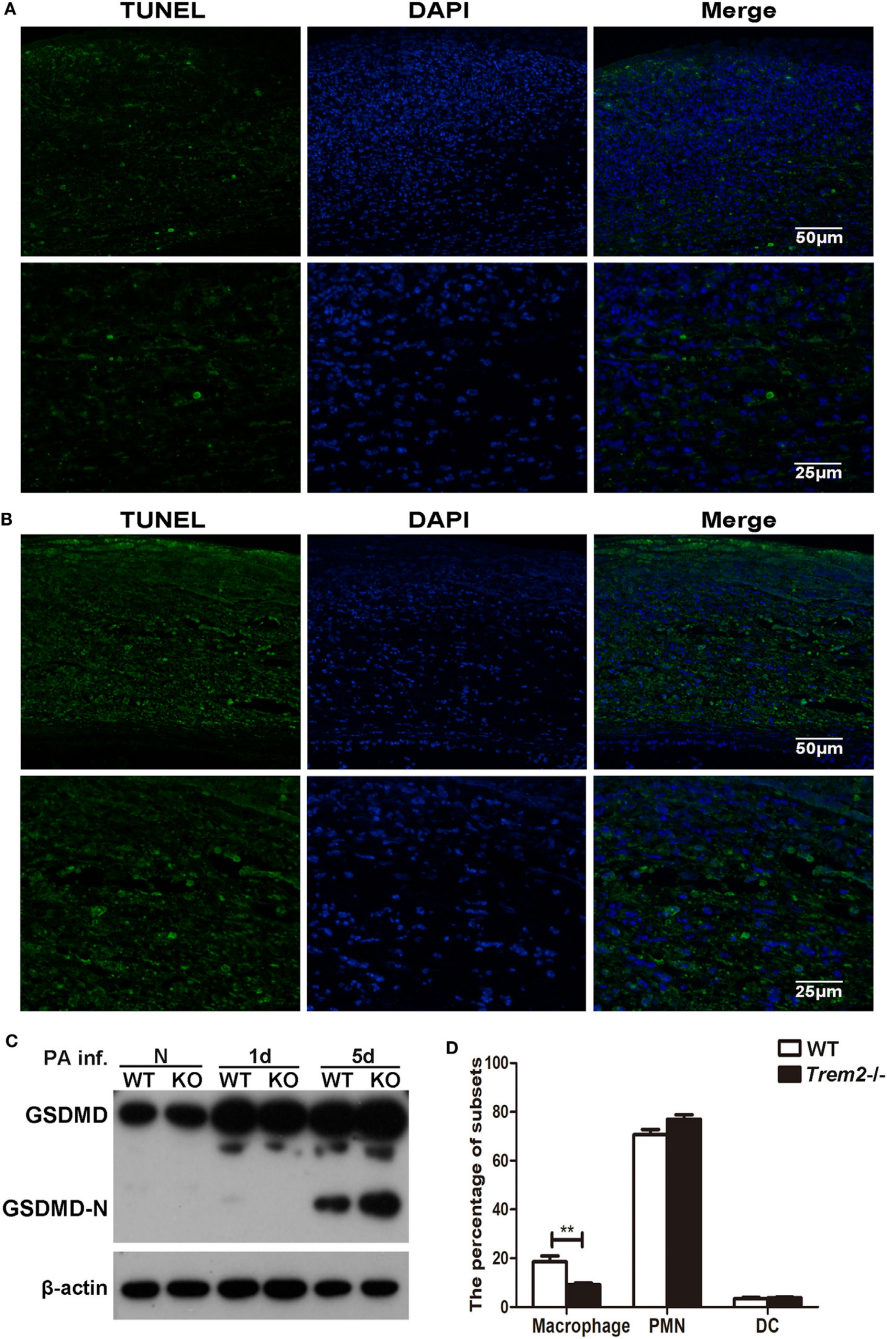
Triggering receptors expressed on myeloid cells 2 (TREM-2) inhibited pyroptosis of infiltrating cells in *Pseudomonas aeruginosa* keratitis. Pyroptosis in the infected cornea assessed with terminal deoxynucleotidyl transferase-mediated uridine 5′-triphosphate-biotin nick end labeling (TUNEL) staining. TUNEL-positive staining (green) was detected in *Trem2*^−/−^ **(A)** versus wild type (WT) **(B)** corneas at 5 days after infection. Cell nuclei were stained with 4,6-diamino-2-phenyl indole (DAPI; blue). Magnification was 200 and 400× respectively. **(C)** The protein levels of GSDMD and its N-terminal domain in *Trem2*^−/−^ versus WT B6 corneas were detected with western blot. **(D)** The percentage of macrophages, polymorphonuclear neutrophils, and dendritic cells were detected by flow cytometry in the infected WT and *Trem2*^−/−^ corneas at 5 days post infection. Data were shown to represent one of three individual experiments each with five mice per group.

Since caspase-1 activation is often associated with cell pyroptosis (a type of pro-inflammatory programmed cell death), here we also assessed cell death with TUNEL staining in WT and *Trem2*^−/−^ corneas after *P. aeruginosa* infection. In contrast to the WT group (Figure 4A), the TREM2-deficient corneas (Figure 4B) showed more intense TUNEL-positive staining in the cornea stroma (magnification = 200×, 400×) after infection, suggesting that TREM2 suppressed the death of the infiltrating cells in *P. aeruginosa*-infected corneas. To confirm whether TREM2-modulated cell death is pyroptosis, we further detected the GSDMD and its N-terminal domain, which was recently identified as downstream of caspase-1 to cause pyroptosis. Western blot results showed that deficiency of TREM2 attenuated cleavage of GSDMD (Figure 4C), suggesting that TREM2 specifically suppressed *P. aeruginosa*-induced pyroptosis. Moreover, our results revealed that the number of macrophages in *Trem2*^−/−^ corneas was lower than that in WT corneas after *P. aeruginosa* infection (Figure 4D), which is consistent with the *in vivo* data showing that TREM2 suppressed caspase-1-dependent pyroptosis in macrophages after *P. aeruginosa* infection (Figures 3D,E). Collectively, these results demonstrated that TREM2 inhibits caspase-1 activation and subsequent pyroptosis in *P. aeruginosa* keratitis.

### Caspase-1 Was Essential for Cornea Pathology Develop and Bacterial Clearance in *P. aeruginosa* Keratitis

**Figure 5:**
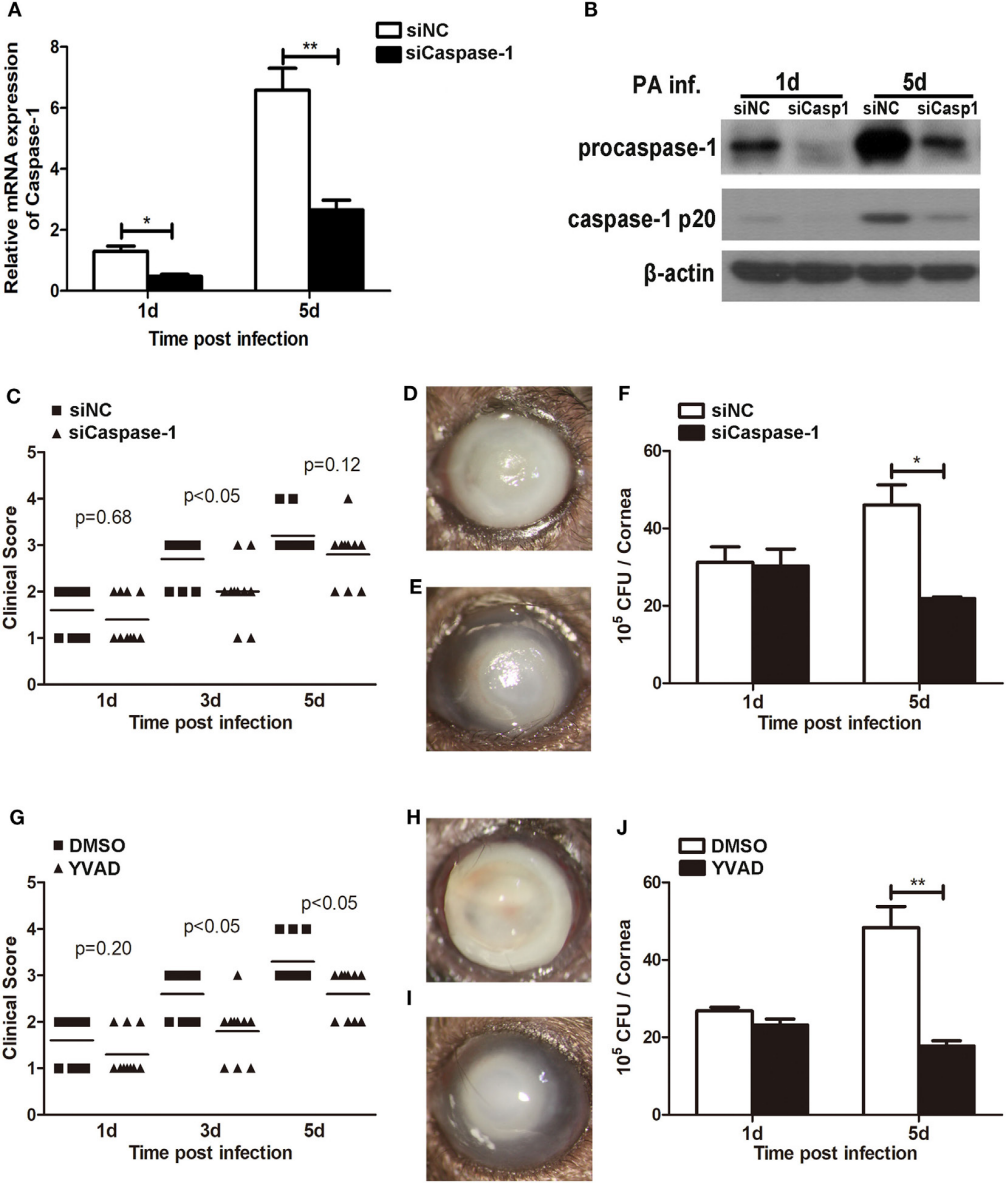
Caspase-1 was essential for disease progression and bacterial clearance in *Pseudomonas aeruginosa* keratitis. **(A–F)** C57BL/6 mice were subconjunctivally injected with caspase-1 siRNA or scrambled control, and then infected with PA routinely. **(G–J)** C57BL/6 mice were subconjunctivally injected with the caspase-1 inhibitor Ac-YVAD-CMK (YVAD) or vehicle control (DMSO), and then infected with *P. aeruginosa* routinely. **(A)** mRNA levels of caspase-1 was examined by real-time PCR in caspase-1 siRNA- versus control-treated corneas at 1 and 5 days after infection. **(B)** Protein levels of caspase-1 were detected with western blot in caspase-1 siRNA- versus control-treated corneas at 1 and 5 days after infection. **(C,G)** Clinical score was recorded for each cornea at 1, 3, and 5 days after infection. Representative photographs of infected eyes in caspase-1 siRNA- **(E)** versus control- **(D)**, YVAD **(I)** versus DMSO **(H)** treated mice were taken at 5 days after infection. **(F,J)** Bacterial load in the infected corneas was examined by plate count assay at 1 and 5 days after infection. Data are the mean ± SEM and represent three individual experiments each with 10 mice per group **(C–E,G–I)** or five mice per group **(A,B,F,J)**. *P < 0.05; **P < 0.01.

We have showed above that activation of caspase-1 and pyroptosis were markedly enhanced in *Trem2*^−/−^ mice in response to *P. aeruginosa* ocular infection (Figures 3 and 4). To further determine whether TREM2 modulated host defense against *P. aeruginosa* through regulating caspase-1 activation and pyroptosis, C57BL/6 mice were subconjunctivally injected with caspase-1 siRNA or caspase-1-specific inhibitor YVAD, followed by routine *P. aeruginosa* corneal infection. The efficacy of silencing was confirmed by both mRNA (Figure 5A) and protein levels (Figure 5B) of caspase-1 which were decreased in the siCaspase-1-treated corneas after *P. aeruginosa* infection. Indeed, clinical scores showed that siCaspase-1 and YVAD-treated B6 corneas exhibited less disease severity (Figures 5C,G). Consistently, knockdown or inhibition of caspase-1 markedly suppress disease severity as demonstrated by less corneal opacity exhibited in the infected corneas (Figures 5E,I) compared with their control group (Figures 5D,H). Furthermore, silencing or inhibiting caspase-1 also resulted in reduced bacterial burden (Figures 5F,J). These data suggested that caspase-1 was required for corneal inflammation and bacterial elimination in *P. aeruginosa* keratitis.

### TREM-2 Promoted Host Resistance Against *P. aeruginosa* Keratitis by Inhibiting Caspase-1-Dependent Pyroptosis

**Figure 6:**
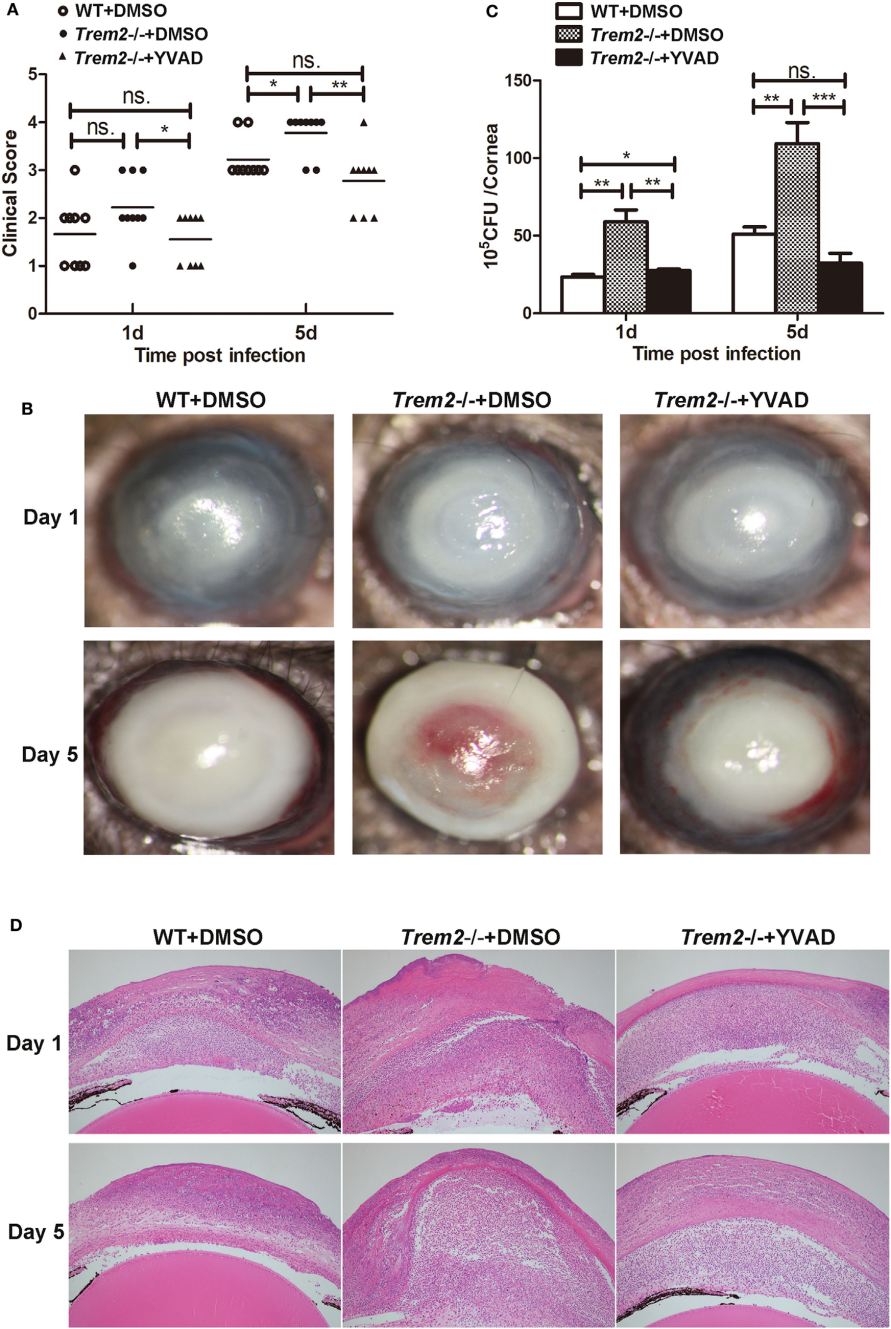
Triggering receptors expressed on myeloid cells 2 (TREM-2) decreased corneal inflammation and bacterial load in *Pseudomonas aeruginosa* keratitis *via* inhibiting caspase-1. Wild type (WT) mice were subconjunctivally injected with the vehicle control (DMSO) and *Trem2*^−/−^ mice were subconjunctivally injected with the vehicle control (DMSO) or caspase-1 inhibitor YVAD, and then infected with *P. aeruginosa* routinely. **(A)** Clinical score was recorded for each cornea at 1 and 5 days after infection. **(B)** Representative photographs of infected eyes in YVAD- versus DMSO-treated *Trem2*^−/−^ mice as well as DMSO-treated WT mice were taken at 1 and 5 days after infection. **(C)** Bacterial load in the infected corneas was examined by plate count in YVAD- versus DMSO-treated *Trem2*^−/−^ corneas as well as DMSO-treated WT corneas at 1 and 5 days after infection. **(D)** Hematoxylin and eosin staining was used to examine the histopathology of infected eyes in DMSO-treated WT (left), DMSO-treated *Trem*2^−/−^ (middle), and YVAD-treated *Trem*2^−/−^ (right) mice at 1 and 5 days after infection. Magnification = 100×. Data are the mean ± SEM and represent three individual experiments each with 10 mice per group **(A,B)** or five mice per group **(C,D)**. *P < 0.05; **P < 0.01; ***P < 0.001.

**Figure 7:**
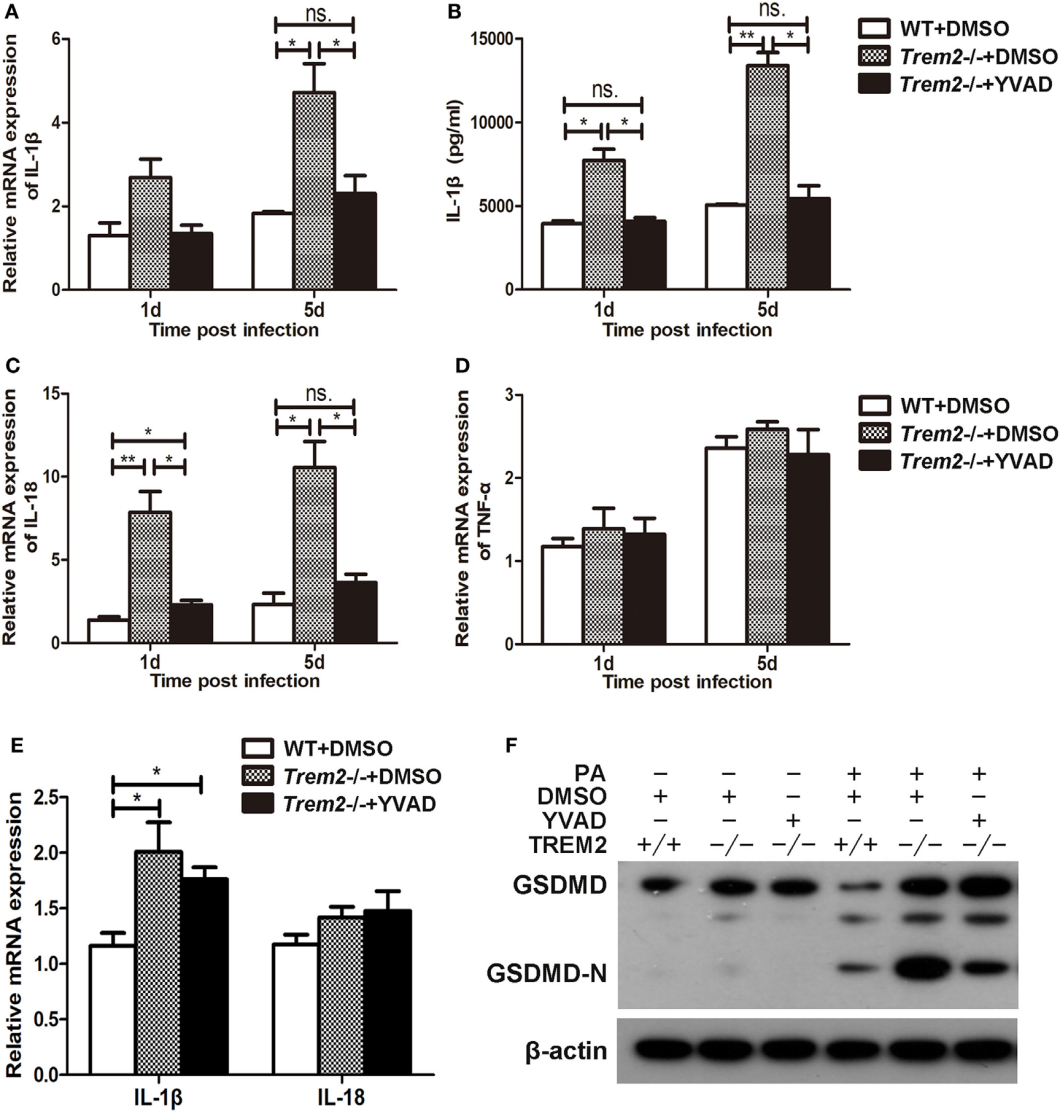
Triggering receptors expressed on myeloid cells 2 (TREM-2) promoted host resistance against *Pseudomonas aeruginosa* keratitis *via* inhibiting caspase-1-dependent pyroptosis. Wild type (WT) mice were subconjunctivally injected with the vehicle control (DMSO) and *Trem2*^−/−^ mice were subconjunctivally injected with the vehicle control (DMSO) or caspase-1 inhibitor Ac-YVAD-CMK (YVAD), and then infected with *P. aeruginosa* routinely. mRNA expression levels of cytokines, including IL-1β **(A)**, IL-18 **(C)**, TNF-α **(D)** were examined by real-time PCR in the infected corneas at 1 and 5 days after infection. **(B)** Protein level of IL-1β in infected corneas was tested by enzyme-linked immunosorbent assay at 1 and 5 days after infection. **(E)** WT and *Trem2*^−/−^ BMDM were treated with YVAD (40 µM) or DMSO for 1 h, followed by heat-killed *P. aeruginosa* treatment at MOI of 5 for 6 h. mRNA expression levels of IL-1β and IL-18 were examined by real-time PCR. **(F)** The protein levels of GSDMD and its N-terminal domain in corneas were detected by western blot. Data were the mean ± SEM and represent three individual experiments each with five mice per group. *P < 0.05; **P < 0.01.

We showed above that activated caspase-1 and pyroptosis were markedly enhanced in *Trem2*^−/−^ corneas (Figures 3 and 4), and caspase-1 was involved in response to *P. aeruginosa* ocular infection (Figure 5). To determine whether caspase-1-dependent pyroptosis is responsible for the TREM2-mediated host resistance against *P. aeruginosa* keratitis, we treated TREM2-deficient mice with caspase-1 inhibitor YVAD or vehicle control DMSO by subconjunctival injection. We found that the increased disease severity in *Trem2*^−/−^ mice was recovered after treating with YVAD (Figures 6A,B). Consistently, treatment with YVAD also restored the TREM2 deficiency-induced higher level of bacterial load and corneal inflammation (Figures 6C,D). Besides, treatment with YVAD markedly blocked enhanced IL-1β expression and secretion (Figures 7A,B), as well as IL-18 expression (Figure 7C) in *Trem2*^−/−^ mice, but did not affect the expression of TNF-α in corneas of these groups (Figure 7D). However, YVAD treatment failed to restore enhanced IL-1β expression in HK-PA-treated *Trem2*^−/−^ BMDMs (Figure 7E), suggesting the reduced mRNA expression of IL-1β in YVAD-treated *Trem2*^−/−^ corneas is likely due to the decreased bacterial load and corneal inflammation. Furthermore, the enhanced pyroptosis in *Trem2*^−/−^ corneas was also restored after YVAD treatment, as indicated by the change of protein levels of GSDMD N-terminal domain (Figure 7F). Taken together, these data indicated that TREM2 protected *P. aeruginosa*-infected corneas of C57BL/6 mice *via* suppressing caspase-1-dependent pyroptosis.

### TREM-2 Inhibited Caspase-1-Dependent Pyroptosis by Mediating the NLRP3 Inflammasome

**Figure 8:**
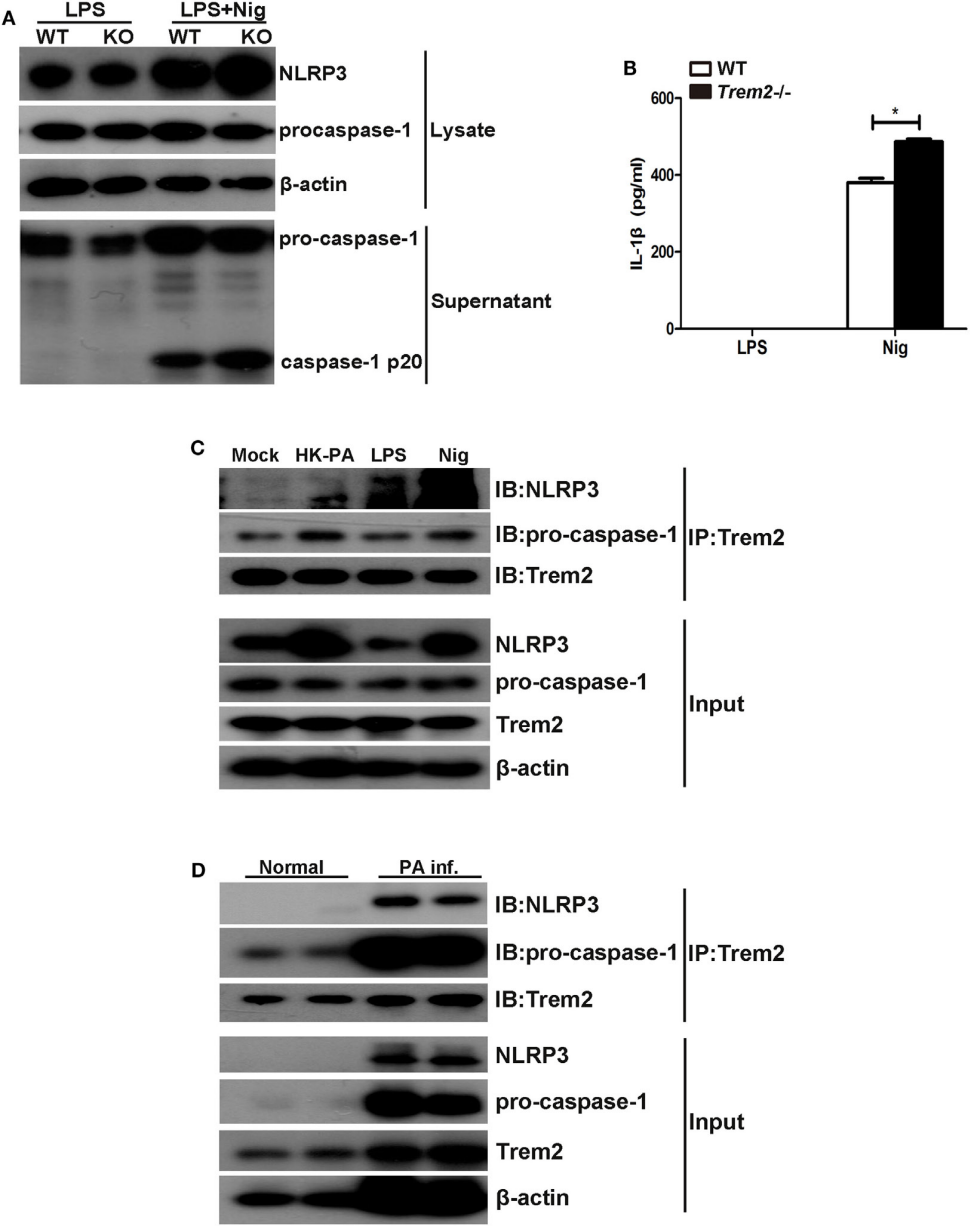
Triggering receptors expressed on myeloid cells 2 (TREM-2) inhibited caspase-1-dependent pyroptosis by mediating the NLRP3 inflammasome. **(A)** Immunoblot analysis of cleaved caspase-1 (p20) in culture supernatants of LPS-primed wild type (WT) and *Trem2*^−/−^ bone marrow-derived macrophages (BMDMs) stimulated with nigericin, immunoblot analysis of the precursors of caspase-1, and NLRP3 in lysates of those cells (Input). **(B)** IL-1β in supernatants from LPS-primed WT and *Trem2*^−/−^ BMDMs stimulated with nigericin were tested by enzyme-linked immunosorbent assay. **(C)** BMDMs from WT C57BL/6 mice were treated with heat-killed *P. aeruginosa* at MOI of 5 for 6 h or stimulated with nigericin, followed by immunoprecipitation using anti-TREM2 antibody. The immunoprecipitates were further immunoblotted with anti-TREM2, anti-caspase-1, and anti-NLRP3 antibodies. The blot shows the detection of caspase-1 and NLRP3 in the TREM2 immunoprecipitates. **(D)** Normal and infected corneas from WT C57BL/6 mice were harvested at 5 days after *Pseudomonas aeruginosa* infection, followed by immunoprecipitation using anti-TREM2 antibody. The immunoprecipitates were further immunoblotted with anti-TREM2, anti-caspase-1, and anti-NLRP3 antibodies. The blot shows the detection of caspase-1 and NLRP3 in the TREM2 immunoprecipitates. Data were the mean ± SEM and represent three individual experiments. *P < 0.05.

As the results showed that TREM2 suppressed caspase-1-dependent pyroptosis in *P. aeruginosa* keratitis, we further investigated the mechanism by which TREM2 mediates caspase-1 activation and cleavage. Since the caspase-1 cleavage as well as the expression of NLRP3 was all upregulated in infected *Trem2*^−/−^ corneas, we first examined the effect of TREM2 on NLRP3 inflammasome activation. We treated LPS-primed WT and *Trem2*^−/−^ BMDMs with nigericin to activate canonical NLRP3 inflammasome, and found that caspase-1 cleavage and IL-1β maturation were indeed promoted in TREM2-deficient BMDMs (Figures 8A,B), suggesting TREM2 may suppress the NLRP3 inflammasome. Moreover, TREM2 co-immunoprecipitated with caspase-1 in BMDMs, and in this complex NLRP3 was also detected when BMDMs were treated with HK-PA or challenged with NLRP3 stimuli (Figure 8C). TREM2 also co-immunoprecipitated with caspase-1 and NLRP3 in *P. aeruginosa*-infected corneas (Figure 8D). Collectively, these results demonstrated that TREM2 inhibits caspase-1-dependent pyroptosis by reducing NLRP3 inflammasome activation.

## Discussion

Triggering receptors expressed on myeloid cells 2 is a novel member of PRRs expressed on myeloid cells, and has been reported to exert a critical role in regulating inflammation response and bacteria elimination (16, 17). However, its regulatory function is still controversial. Studies have demonstrated that TREM2 functions as a negative regulator in the immune response. However, Danese’s group reported that TREM-2 deficient DCs produced lower levels of inflammatory cytokines in a murine model of inflammatory bowel disease (27). Knapp’s lab also found that TREM-2 deficiency improved lung pathology and prevented systemic inflammation during pneumococcal pneumonia (17). These studies suggested a promotive role of TREM2 in regulating inflammatory response. Our previous study demonstrated that TREM2 suppressed *P. aeruginosa*-induced corneal inflammation and decreased bacterial loads in resistant BALB/c mice (which are Th2 responders), but the underlying bactericidal mechanism remains unclear (20). In this study, we found that TREM2 promotes host resistance against *P. aeruginosa* keratitis by inhibiting caspase-1-dependent pyroptosis in susceptible C57BL/6 mice (which are Th1 responders), providing evidence of a novel mechanism of TREM2-mediated immune defense against *P. aeruginosa*.

After infection, microbial pathogens and their virulence factors may impair normal organ function *vi*a inducing host cell death, such as apoptosis, necrosis, autophagy, and pyroptosis. Generally, pathogens may persist in infected hosts by causing the death of cells that required for host defense (28). While some intracellular pathogens may prevent cell death to escape immune clearance and disseminate to other host cells (29). Holtzman’s group demonstrated that the increased TREM2 expression induced by viral replication suppressed apoptosis of lung macrophages, and thus led to chronic inflammation disease in a murine model of Sendai virus infection (18). Colonna’s lab also found that autophagic-like vesicles accumulated in the microglia of TREM2-deficient mice during the development of AD (19). Moreover, Colonna’s lab reported that DAP12, an important adaptor of TREM2, was essential for the proliferation and survival of born marrow-derived macrophages (30). These results provide clues to the correlation between TREM2 and programmed cell death. Unlike viral infection, chronic infection or non-infective diseases, *P. aeruginosa* infection usually induces an acute inflammatory response, such as *P. aeruginosa* keratitis, which often leads to corneal perforation with 48 h postinfection if lacking proper treatment. It is reported that *P. aeruginosa* was able to trigger caspase-1-initiated pyroptosis, which was characterized by increased cell size due to osmotic swelling, and subsequent rupture of the cytoplasmic membrane as well as massive secretion of inflammatory cytokines IL-1β and IL-18 (31, 32). In this study, we found TREM2 deficiency promoted activation of caspase-1, secretion of IL-1β and subsequent cell death in *P. aeruginosa* keratitis. Recently, Shao F’s lab identified GSDMD as downstream of caspase-1 and revealed that after cleavage by caspase-1 and subsequent release of C-terminal domain, the N-terminal domain of GSDMD can accumulate on the plasma membrane to form pores and, therefore, result in pyroptosis (33). Consistently, our results showed that TREM2 deficiency promoted the cleavage of GSDMD to form its N-terminal domain. Besides, caspase-1-specific inhibitor YVAD restored TREM2-deficient-mediated increased cell death and IL-1β secretion. Collectively, these results indicated that TREM2 inhibited caspase-1-dependent pyroptosis induced by *P. aeruginosa* infection. To our knowledge, our study provides the first evidence that TREM2-mediated caspase-1 activation and pyroptosis. In addition, our data showed that caspase-1 activation in macrophage enhanced in infected *Trem2*^−/−^ corneas, while its activation in PMN and DC were comparable in WT and *Trem2*^−/−^ corneas. In the meantime, the number of macrophages was significantly decreased in *Trem2*^−/−^ corneas compared with WT group after *P. aeruginosa* infection. Together, these findings support that the regulatory function of TREM2 in *P. aeruginosa keratitis* is primarily exerted in macrophages.

Pyroptosis was reported to restrict the survival of several intracellular pathogens, including *L. pneumophila* (12) and *F. tularensis* (13). These intracellular pathogens escape from host immune defense through residence and replication within macrophages, whereas pyroptosis exposes them to effective immune environment, lead to the subsequent bacterial phagocytosis and intracellular killing by neutrophils (12). However, the role of pyroptosis in *P. aeruginosa* elimination remains disputable. Prince’s group demonstrated that in a murine model of acute pneumonia, inflammasome activation impaired the ability of alveolar macrophages to eliminate *P. aeruginosa* by promoting macrophage pyroptosis (14). In addition, Hauser’s lab reported that the RhsT protein of *P. aeruginosa* promoted phagocytic cells undergoing inflammasome-mediated cell death with more IL-1β and IL-18 production and in favor of bacterial survival (34). To some extent, these results provide some clues that caspase-1-initiated pyroptosis may suppress *P. aeruginosa* clearance. In this study, we demonstrated that lack of TREM2 promoted bacterial survival, which could be restored by blockage of pyroptosis using caspase-1 inhibitor, suggesting that increased bacteria load is associated with enhanced caspase-1 activation and pyroptosis.

Along with caspase-1-dependent pyroptosis, plenty of pro-inflammatory mediators release from dead cells, such as IL-1β and IL-18. Both IL-1β and IL-18 belong to the IL-1 family cytokines. Similar to IL-1β, IL-18 is synthesized as an inactive precursor and can be processed by caspase-1 to generate an active form. But unlike IL-1β precursor, which is induced by microbial and inflammatory stimuli, the IL-18 precursor is constitutively expressed in most of the human and mouse cells (35). Our data showed that treatment with HK-PA did not induce IL-18 expression and no significant difference in IL-18 expression was detected in HK-PA-treated *Trem2*^−/−^ and WT corneas and BMDMs. In contrast, IL-18 expression was enhanced in mouse corneas and BMDMs after *P. aeruginosa* infection, and TREM2 deficiency increased the PA-induced IL-18 expression *in vivo*. The underlying mechanism of different IL-18 expression in PA infection versus HK-PA treatment remains unknown, which needs further investigation.

*In vivo* studies proved that IL-1β impairs the *P. aeruginosa* clearance by using a murine model of *P. aeruginosa* pneumonia (36). Moreover, IL-1β has been reported as local inflammation mediator in bacterial keratitis (37, 38). IL-1β secretion may be promoted through inflammasome-dependent or -independent pathways. Previously, Hazlett’s group reported that knockout of caspase-1 (which is actually caspase-1 and caspase-11 double knockout), or treatment with caspase-1 inhibitor markedly decreases the ocular inflammation and bacterial load in *P. aeruginosa* keratitis (39, 40). However, Pearlman’s group found that in the *P. aeruginosa*-infected corneas, IL-1β cleavage was dependent on serine proteases of neutrophils but independent of caspase-1 and NLRC4 (41). In our study, TREM2 deficiency resulted in massive secretion of IL-1β which could be restored by treatment with caspase-1-specific inhibitor YVAD. Besides, knockout of TREM2 did not affect TNF-α, which is closely related to cell apoptosis and NF-κB signaling, suggesting TREM2 deficiency may promote IL-1β production in a caspase-1-dependent manner. To be noted, YVAD treatment blocked IL-1β and IL-18 transcription in *Trem2*^−/−^ corneas but failed to restore enhanced IL-1β expression in HK-PA treated *Trem2*^−/−^ BMDMs. Our data showed that inhibition of caspase-1 decreased bacterial load and inflammatory cell infiltration in the infected corneas. Since bacterial virulence factors can activate local and infiltrated inflammatory cells to induce the IL-1β and IL-18 production, we speculated the decreased bacterial load and corneal inflammation may be the reason of reduced IL-1β and IL-18 mRNA levels in YVAD-treated *Trem2*^−/−^ corneas.

It is worth to mention that, in our previous study, silencing of TREM2 in corneas of BALB/c mice increased TNF-α expression, whereas in this study, lack of TREM2 failed to upregulate TNF-α in C57BL/6 mice. We also established a murine model of *P. aeruginosa*-induced sepsis using TREM2-deficient versus WT C57BL/6 mice, and found that TNF-α expression was comparable between the two groups (data not shown), which is consistent with our observation in this study. A recent study revealed that Th1 cells have more detectable amounts of NLRP3 protein than Th2 cells (42), suggesting the inflammasome activation may be different between these T helper subsets. Since C57BL/6 and BALB/c mice are known to preferentially demonstrate Th1 and Th2 responses, respectively, we speculate that the different effects of TREM2 on TNF-α expression in C57BL/6 and BALB/c mice may result from distinct responses (Th1- or Th2-favored).

Our *in vivo* studies indicated that TREM2 deficiency not only increased the production of IL-1β and IL-18, but also promoted expression of MIP-2. In susceptible C57BL/6 mice, IL-1β is able to upregulate the production of MIP-2, an important chemokine which attracts PMN influx into the infected cornea and, therefore, contributes to irreversible corneal tissue destruction (37). In this regard, the increased MIP-2 and severe inflammation are probably due to the high level of IL-1β in TREM2-deficient mice.

Caspase-1-dependent pyroptosis often results from activation of inflammasomes (43–45). Many Studies have demonstrated that *P. aeruginosa* infection mainly activated the NLRC4 inflammasome by its flagella. Recently, Hazlett’ group reported that the NLRC4 inflammasome in CD11b^low^Ly6G^low^ cells contributed to the host resistance of BALB/c mice in response to *P. aeruginosa* infection, through regulating caspase-1 and IL-1β production (46). However, our previous research in human macrophages indicated that *P. aeruginosa* triggered the assembly of the NLRP3 inflammasomes, which suppressed the killing of *P. aeruginosa* by triggering autophagy of macrophages (47). Our study showed that caspase-1 cleavage and IL-1β maturation were promoted in TREM2-deficient BMDMs after treated with canonical NLRP3 stimuli nigericin (Figures 8A,B), suggesting TREM2 suppressed NLRP3 inflammasome to inhibit caspase-1 activation and pyroptosis. After *P. aeruginosa* infection, other inflammasomes (such as NLRC4 inflammasome or caspase-8/caspase-11-mediated noncanonical inflammasome) may also be activated. Loss of TREM2 delayed but did not halt the disease progression. Most of the wild type corneas were perforated at 5 days postinfection, while corneas of TREM2-deficient animals were perforated at 3 days postinfection.

Our endogenous Co-IP data showed that, TREM2 co-immunoprecipitated with procaspase-1 and NLRP3 in BMDMs treated with HK-PA, LPS, or nigericin, as well as *P. aeruginosa*-infected mouse corneas (Figures 8C,D). Since TREM2 is a transmembrane receptor with surface and cytoplasmic domains, the coimmunoprecipitation of NLRP3 with TREM2 suggests that TREM2 may regulate inflammasome activation by direct interaction.

In summary, we demonstrated that TREM2 deficiency increased host susceptibility to *P. aeruginosa* corneal infection in C57BL/6 mice. The increased sensitivity in *Trem2*^−/−^ mice was associated with upregulated NLRP3 inflammasome activation and subsequent production of pro-inflammatory cytokines IL-1β. Furthermore, our data showed that caspase-1-dependent pyroptosis was responsible for more serious tissue pathological injuries and increased bacterial load. Together, our study revealed a novel mechanism by which TREM2 mediates the immune defense against *P. aeruginosa*.

## Ethics Statement

The procedure was performed in accordance with the National Commission for the Protection of Subjects of Biomedical and Behavioral Research guidelines for animal experiments. All efforts were made to minimize suffering. Before the study was initiated, all experiment protocols were approved by the local Ethics Committee of Sun Yat-sen University.

## Author Contributions

MW and WQ wrote the manuscript. MW designed experiments. WQ, YW, YW, YL, KC, XL, and ZZ performed experiments and analyzed data. XH provided scientific expertise. MW supervised the project.

## Conflict of Interest Statement

The authors declare that the research was conducted in the absence of any commercial or financial relationships that could be construed as a potential conflict of interest.

## Supplementary Material

Figure S1 **(A)** mRNA expression level of caspase-1 was examined by real-time PCR in heat-killed *Pseudomonas aeruginosa* (HK-PA) treated wild type (WT) and *Trem2*^−/−^ B6 corneas at 5 days after treatment and WT and *Trem2*^−/−^ BMDM which were treated with HK-PA at MOI of 5 for 6 h. **(B)** The protein level of caspase-11 in *P. aeruginosa*-infected WT and *Trem2*^−/−^ B6 corneas were detected with western blot at 1 and 5 days after infection. **(C)** A6(1) cells were transfected with TREM2 siRNA versus control scrambled siRNA, followed by HK-PA treatment at MOI of 5 for 6 h. mRNA expression levels of TREM2, IL-1β, IL-18, MIP-2, and caspase-1 was examined by real-time PCR. Data were the mean ± SEM and represent three individual experiments. **P* < 0.05.
