# Supplementary figures and images for: Triggering Receptors Expressed on Myeloid Cells 2 Promotes Corneal Resistance Against Pseudomonas aeruginosa by Inhibiting Caspase-1-Dependent Pyroptosis
Source: Front Immunol. 2018 May 25;9:1121. doi: 10.3389/fimmu.2018.01121 (PMC5980993; doi:10.3389/fimmu.2018.01121)

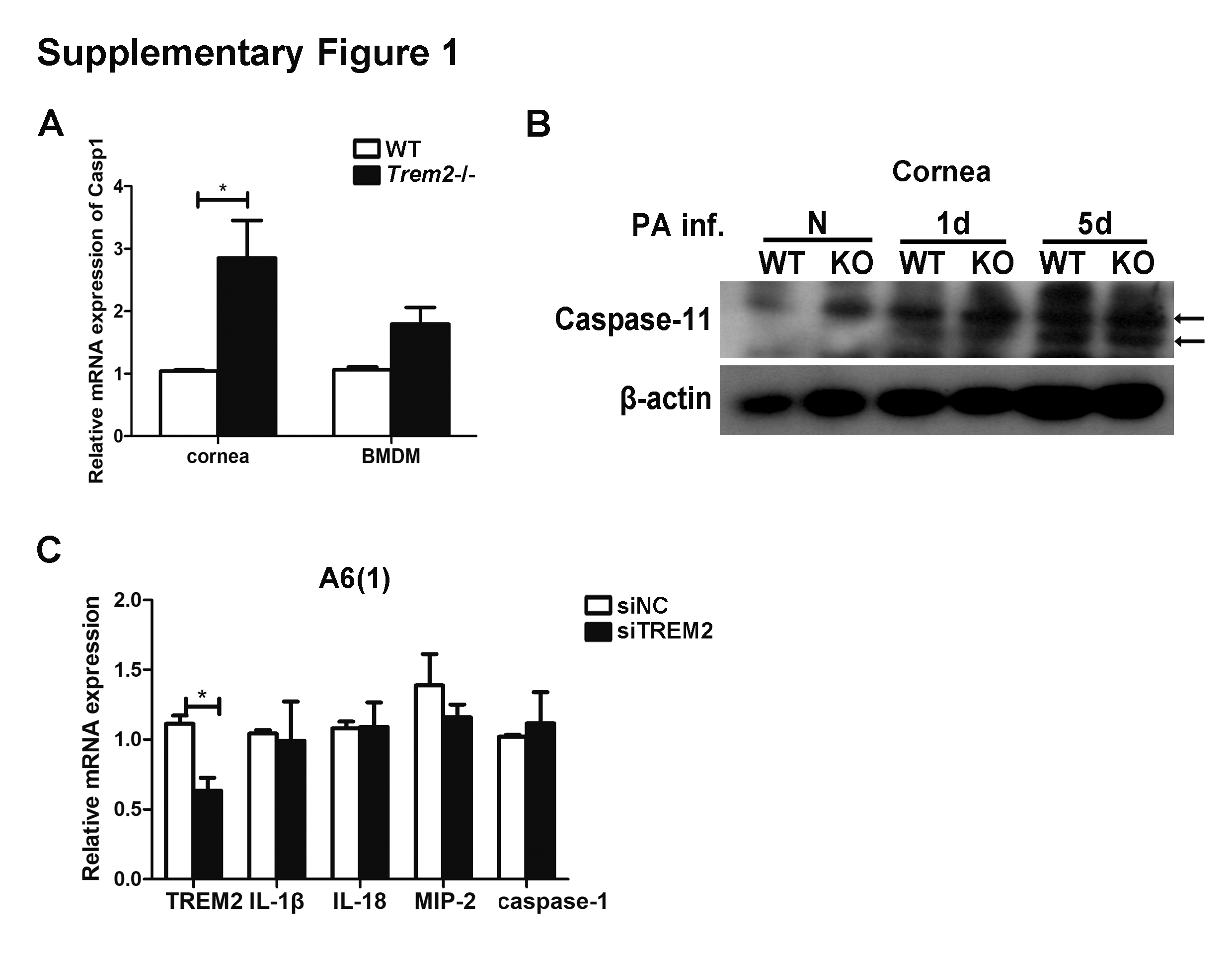

Supplement: Figure S1 — (A) mRNA expression level of caspase-1 was examined by real-time PCR in heat-killed Pseudomonas aeruginosa (HK-PA) treated wild type (WT) and Trem2−/− B6 corneas at 5 days after treatment and WT and Trem2−/− BMDM which were treated with HK-PA at MOI of 5 for 6 h. (B) The protein level of caspase-11 in P. aeruginosa-infected WT and Trem2−/− B6 corneas were detected with western blot at 1 and 5 days after infection. (C) A6(1) cells were transfected with TREM2 siRNA versus control scrambled siRNA, followed by HK-PA treatment at MOI of 5 for 6 h. mRNA expression levels of TREM2, IL-1β, IL-18, MIP-2, and caspase-1 was examined by real-time PCR. Data were the mean ± SEM and represent three individual experiments. *P < 0.05. [file image_1.tif]
