# Supplementary material for: Triggering Receptors Expressed on Myeloid Cells 2 Promotes Corneal Resistance Against Pseudomonas aeruginosa by Inhibiting Caspase-1-Dependent Pyroptosis
Source: Front Immunol. 2018 May 25;9:1121. doi: 10.3389/fimmu.2018.01121 (PMC5980993; doi:10.3389/fimmu.2018.01121)
Supplement: Supplementary file 2 [file table_1.docx]

**Table S1.** Nucleotide sequences of the speciﬁc primers used in PCR ampliﬁcation

| Gene | Primer Sequence (5'-3') |  |
| --- | --- | --- |
| *IL-1β* | CGC AGC AGC ACA TCA ACA AGA GC  TGT CCT CAT CCT GGA AGG TCC ACG | F  R |
| *IL-18* | GCC TGT GTT CGA GGA TAT GAC TGA  TTC ACA GAG AGG GTC ACA GCC A | F  R |
| *TNF-α* | CAC AGA AAG CAT GAT CCG CGA C  TGC CAC AAG CAG GAA TGA GAA GAG | F  R |
| *IFN-γ* | CTCTGAGACAATGAACGCTAC  TTTCTTCCACATCTATGCCAC | F  R |
| *MIP-2* | TGT CAA TGC CTG AAG ACC CTG CC  AAC TTT TTG ACC GCC CTT GAG AGT GG | F  R |
| *β-actin* | GATTACTGCTCTGGCTCCTAGC  GACTCATCGTACTCCTGCTTGC | F  R |
